# Pharmacogenetics Based Dose Prediction Model for Initial Tacrolimus Dosing in Renal Transplant Recipients

**DOI:** 10.3389/fphar.2021.726784

**Published:** 2021-11-30

**Authors:** Lekshmy Srinivas, Noble Gracious, Radhakrishnan R. Nair

**Affiliations:** ^1^ Laboratory Medicine and Molecular Diagnostics, Rajiv Gandhi Centre for Biotechnology, Thiruvananthapuram, India; ^2^ Department of Nephrology, Government Medical College, Thiruvananthapuram, India

**Keywords:** pharmacogenetics, tacrolimus, dose prediction model, *CYP3A5*, renal transplantation, NODAT, rejection

## Abstract

Tacrolimus, an immunosuppressant used in solid organ transplantation, has a narrow therapeutic index and exhibits inter-individual pharmacokinetic variability. Achieving and maintaining a therapeutic level of the drug by giving appropriate doses is crucial for successful immunosuppression, especially during the initial post-transplant period. We studied the effect of *CYP3A5*, *CYP3A4*, and *ABCB1* gene polymorphisms on tacrolimus trough concentrations in South Indian renal transplant recipients from Kerala to formulate a genotype-based dosing equation to calculate the required starting daily dose of tacrolimus to be given to each patient to attain optimal initial post-transplant period drug level. We also investigated the effect of these genes on drug-induced adverse effects and rejection episodes and looked into the global distribution of allele frequencies of these polymorphisms. One hundred forty-five renal transplant recipients on a triple immunosuppressive regimen of tacrolimus, mycophenolate mofetil, and steroid were included in this study. Clinical data including tacrolimus daily doses, trough levels (C_0_) and dose-adjusted tacrolimus trough concentration (C_0_/D) in blood at three time points (day 6, 6 months, and 1-year post-transplantation), adverse drug effects, rejection episodes, serum creatinine levels, etc., were recorded. The patients were genotyped for CYP3A5*3, CYP3A4*1B, CYP3A4*1G, *ABCB1* G2677T, and *ABCB1* C3435T polymorphisms by the PCR-RFLP method. We found that CYP3A5*3 polymorphism was the single most strongly associated factor determining the tacrolimus C_0_/D in blood at all three time points (*p* < 0.001). Using multiple linear regression, we formulated a simple and easy to compute equation that will help the clinician calculate the starting tacrolimus dose per kg body weight to be administered to a patient to attain optimal initial post-transplant period tacrolimus level. *CYP3A5* expressors had an increased chance of rejection than non-expressors (*p* = 0.028), while non-expressors had an increased risk for new-onset diabetes mellitus after transplantation (NODAT) than expressors (*p* = 0.018). Genotype-guided initial tacrolimus dosing would help transplant recipients achieve optimal initial post-transplant period tacrolimus levels and thus prevent the adverse effects due to overdose and rejection due to inadequate dose. We observed inter-population differences in allele frequencies of drug metabolizer and transporter genes, emphasizing the importance of formulating population-specific dose prediction models to draw results of clinical relevance.

## Introduction

Tacrolimus, a calcineurin inhibitor immunosuppressant used in solid organ transplant recipients, exhibits interindividual pharmacokinetic variability that affects the dose required to reach the target concentration in blood (Coto et al., 2011). Attaining and sustaining a therapeutic level of the immunosuppressants by administering appropriate doses is crucial, particularly during the initial post-transplant period. The success of a transplant depends on a fragile balance between immunosuppression and rejection (Provenzani et al., 2013). Due to the narrow therapeutic window of tacrolimus, therapeutic drug monitoring (TDM) is essential to maintain adequate blood concentrations to prevent graft rejection due to inadequate immunosuppression and toxicity due to higher drug levels (Brunet et al., 2019). Despite the advances in medicine, attaining and maintaining the optimal therapeutic range of tacrolimus specific to different post-transplant time points remains a challenge (Herrero et al., 2013; Ben-Fredj et al., 2020).

Tacrolimus is metabolized by the Cytochrome P450 3A5 and 3A4 enzymes (CYP3A5 and CYP3A4) in the gut and liver, and transported in the gut by an efflux pump, P-glycoprotein (P-gp) (Sakaeda et al., 2003; De Jonge et al., 2012). *CYP3A5* and *CYP3A4* genes are part of a cluster of Cytochrome P450 genes on the long arm of chromosome 7 (7q21.1) (Li et al., 2014). The ATP Binding Cassette Subfamily B Member 1 (*ABCB1*) or Multi-Drug Resistance 1 (*MDR1*) gene, which codes for P-gp, is also located nearby (7q21.12) (Chen et al., 1990). Polymorphisms in *CYP3A5*, *CYP3A4*, and *ABCB1* genes could have important roles in tacrolimus blood concentration and dose requirement (Haufroid et al., 2006; Mourad et al., 2006; Tamashiro et al., 2017). The gene expression and enzyme activity of CYP3A5 depends mainly on the CYP3A5*3 polymorphism (6986G > A, rs776746) located in the intron 3. A nucleotide change from A to G creates a cryptic splice site, which causes altered mRNA splicing resulting in a premature termination codon and hence a non-functional protein (Kuehl et al., 2001). Individuals with the CYP3A5*3/*3 genotype are considered to be *CYP3A5* non-expressors. *CYP3A4* gene expression is regulated by CYP3A4*1B and CYP3A4*1G polymorphisms. The promoter polymorphism CYP3A4*1B (-392A > G, rs2740574) may be associated with enhanced CYP3A4 expression owing to reduced binding of a transcriptional repressor (Amirimani et al., 2003). In the case of CYP3A4*1G, the G to A substitution at IVS10 + 12 is correlated with an increased transcription of the *CYP3A4* gene (He et al., 2011). A missense mutation in exon 21 of *ABCB1* gene, G2677T, results in an Ala to Ser amino acid change at position 893 of the protein, and has been associated with altered P-gp expression (Seven et al., 2014). The *ABCB1* C3435T (I1145I) is a synonymous polymorphism which has been shown to correlate with the expression levels and function of P-gp. In *ABCB1* C3435T polymorphism, there is a C to T substitution at nucleotide position 3435 in exon 26. Although it does not change its encoded amino acid with Ile at position 114522, it can affect the post-transcriptional processing of mRNA or affect the process of alternative transcript splicing (Tamura et al., 2012).

Though many studies have been carried out concentrating on the role of *CYP3A5* on tacrolimus blood levels, the pharmacogenetic factors identified so far were of insufficient predictive value and not much of clinical use (Coto et al., 2011; Boughton et al., 2013; Chen and Prasad, 2018).

There is a lack of data from South Indian patients on the effect of multiple genes on tacrolimus trough concentrations in the early post-transplantation period. This study was carried out to investigate the effect of *CYP3A5*, *CYP3A4*, and *ABCB1* genes on dose-adjusted tacrolimus trough concentrations in South Indian renal transplant recipients from Kerala. The study aimed to build a pharmacogenetics-based dosing equation to calculate the required starting daily dose to be administered to each patient to attain optimal initial post-transplant period tacrolimus level based on his genotype. Genotype-guided dosing, rather than dosing based solely on the patient’s body weight, maybe a preferred strategy to determine the initial dose of tacrolimus in patients undergoing solid organ transplantation. This would help prevent the adverse effects of overdose and transplant rejection due to inadequate dose. We also looked into the association of the selected gene polymorphisms with drug-induced adverse effects and rejection episodes. In addition, we analyzed the global variation in distribution of these polymorphisms by comparing their allelic frequencies in our population with the other world populations allele frequencies data.

## Materials and Methods

### Study Subjects

For this prospective study, 156 renal transplant recipients belonging to an ethnically matched Malayalam-speaking population of Kerala, South India, receiving tacrolimus as an immunosuppressant were recruited from the Department of Nephrology, Government Medical College, Thiruvananthapuram. All 156 patients were genotyped. Since follow-up data were available only for 145 patients, further analyses were performed only using these patients. Patients who were more than 15 years of age and less than 60 were included in the study. Patients receiving mTOR inhibitors (sirolimus, everolimus) along with tacrolimus, or medications known to influence drug levels (diltiazem, fluconazole) were excluded from the study. Patients with delayed graft function and early graft dysfunction within 1-week post-transplantation were excluded from the association analysis of genetic polymorphisms with dose-adjusted tacrolimus trough concentration (C_0_/D) since the treatment modalities for these might affect tacrolimus levels and thus, the study results.

The study was approved by the Human Ethics Committees of Rajiv Gandhi Centre for Biotechnology and Government Medical College, Thiruvananthapuram. Informed, written consent in a standard consent form was obtained from all the study subjects to participate in the study after being provided with a full explanation of study protocols, objectives, benefits, and risks.

All patients were on a triple immunosuppressive regime [tacrolimus, mycophenolate mofetil (MMF), and steroid]. All study participants received an initial tacrolimus starting dose of 0.075–0.1 mg/kg body weight per day in 2 divided doses from day minus 2 of transplantation as per the institutional protocol. The dose was then adjusted to achieve a target tacrolimus trough concentration (C_0_) of 7–10 ng/ml for first 3 months of transplantation.

### Sample Size Estimation

Sample size and statistical power were calculated by one-sample *t*-test using nQuery Sample Size Software (Statistical Solutions, Cork, Ireland) by comparing mean tacrolimus drug levels in the whole population vs. patients with specific genotypes in *CYP3A5*, *ABCB1* (Vattam et al., 2013) and *CYP3A4* (Li et al., 2014). The required sample size to study the effects of *CYP3A5*, *CYP3A4*, and *ABCB1* polymorphisms on the dose-adjusted tacrolimus level (with 80% power and test significance level, *α* = 0.05) was 137.

### Clinical Data Collection

Patient demographic characteristics like age of the patient during transplant, gender, body weight, etc., were recorded. Data including tacrolimus daily doses (mg) and trough levels C_0_ (ng/ml) in blood on day 6, 6 months, and 1-year post-transplantation, concomitant medications and events including rejection and adverse drug effects like tacrolimus toxicity, NODAT and post-transplant erythrocytosis, serum creatinine levels and all lab investigation results were collected from the medical record during the follow up visits. Liquid chromatography coupled to tandem mass spectrometry (LC-MS/MS) method was used to determine tacrolimus concentration in blood. The daily tacrolimus dose was noted, and the weight-adjusted tacrolimus dose was calculated using daily tacrolimus dose/weight (mg/kg per day). The dose-adjusted tacrolimus trough concentration (C_0_/D) was calculated by dividing the measured C_0_ by the corresponding daily weight-adjusted tacrolimus dose (ng/ml per mg/kg).

### DNA Isolation and Genotyping

We collected 5 ml of peripheral blood from the patients in K_2_-EDTA coated Vacutainer^®^ for DNA isolation. Genomic DNA was isolated using the DNA Isolation Kit for Mammalian Blood (Roche Life Science, United States).

The patients were genotyped for 5 SNPs from *CYP3A5* (*3/rs776746), *CYP3A4* (*1B/rs2740574 and *1G/rs2242480) and *MDR1* or *ABCB1* genes (Ex22 G2677T/rs2032582 and Ex27 C3435T/rs1045642). Genes and SNPs were selected based on their functional significance and previous reports on association with tacrolimus concentrations.

Genotyping was performed by the polymerase chain reaction-restriction fragment length polymorphism (PCR-RFLP) method. PCR amplification was carried out using specific primers in the Applied Biosystems^®^ Veriti^®^ or Eppendorf^®^ Mastercycler^®^ thermal cyclers. PCR was done in a final volume of 10 µL. PCR reaction mix consisted of 100 ng of genomic DNA, 5 pmol each of forward and reverse primers, and 1X EmeraldAmp^®^ PCR Master Mix (Clontech Laboratories, Inc.). Cycling parameters were initial denaturation at 94°C for 1 min, annealing for 30 s, extension at 72°C for 20 s, and a final extension step at 72°C for 1 min; the number of cycles was 30. The amplified PCR products was digested with 5 Units of the respective restriction enzyme (NEB Inc., United States and ThermoFisher Scientific, United States) and a specific 1X buffer in a final volume of 15 µL. PCR primers, annealing temperature, restriction enzymes, amplicon and allele sizes for each polymorphism are summarized in Supplementary Table S1.

### Statistical Analysis

Quantitative variables were summarized using mean and standard deviation. Categorical variables were represented using frequency and percentage. Independent sample *t*-test and ANOVA were used for comparing continuous variables between groups. Pearson Chi-square test was used for comparing categorical variables between groups. Linear regression was used to find out independent predictors of tacrolimus C_0_/D ratio**.** All statistical analyses were performed using the SPSS^®^ statistical software package (version 22.0, IBM Inc., Armonk, NY, United States). A *p*-value of <0.05 was considered statistically significant. Pair-wise Linkage disequilibrium (LD) between SNPs was calculated using UNPHASED software for genetic association analysis, version 3.1.7. Deviations from the Hardy-Weinberg equilibrium (HWE) were tested for all polymorphisms by comparing observed and expected genotype frequencies using the miniPCR Hardy-Weinberg calculator spreadsheet (https://www.minipcr.com/wp-content/uploads/miniPCR-Hardy-Weinberg-Calculator.xlsx). The global variation in distribution of the polymorphisms was analyzed by comparing their allelic frequencies in our population with the 1000 genomes browser Phase 3 populations allele frequencies data (https://www.ncbi.nlm.nih.gov/variation/tools/1000genomes/) downloaded from the Ensembl database (https://asia.ensembl.org/index.html). A description of 1000 Genomes Project Phase 3 populations used for comparison in the present study is given in Supplementary Table S2.

## Results

The baseline characteristics of the study population are given in Table 1. Tacrolimus dosing and trough concentrations of the patients at three time points (6^th^ day, 6 months, and 1 year after transplantation) are listed in Table 2. The genotype frequencies of all the SNPs were similar to those expected under Hardy-Weinberg equilibrium (*p* > 0.05). The observed genotype and allele frequencies of the polymorphisms are presented in Table 3.

**TABLE 1:** Baseline characteristics of the study population.

**Number of patients (N)**	145
**Age (years) (mean ± SD)**	36.61 ± 10.58
**Gender [male (%)/female (%)]**	118(81.4)/27 (18.6)
**Type of donor (*n* = 141) n (%)**	
Live	110(78)
Cadaver	31(22)
**Native kidney disease n (%)**	
Chronic glomerulonephritis	82(56.6)
Reflux nephropathy	11(7.6)
Diabetic nephropathy	7(4.8)
Others	22(15.2)
Unknown	23(15.9)
**Induction n (%)**	
Basiliximab	20(13.8)
ATG	20(13.8)
Rituximab	7(4.8)
None	98(67.6)

**TABLE 2:** Tacrolimus dosing in the study population at 3 time points.

	6th day	6 months	1 year
	(*n* = 139)	(*n* = 89)	(*n* = 66)
Bodyweight (kg) (mean ± SD)	59.55 ± 12.82	62.98 ± 12.58	63.63 ± 12.81
Tacrolimus dose (mg/day)	3.88 ± 1.17	3.58 ± 1.16	3.38 ± 1.21
Tacrolimus concentration (ng/ml)	7.11 ± 3.99	6.79 ± 2.5	6.68 ± 2.64
Weight adjusted tacrolimus dose (mg/kg/day)	0.06 ± 0.02	0.05 ± 0.02	0.05 ± 0.02
Concentration/Dose ratio (C_0_/D) (ng/ml)/(mg/kg)	113.87 ± 60.42	132.47 ± 69.65	139.44 ± 70.2

**TABLE 3:** Genotype and allele frequencies of SNPs in the study population.

Polymorphism	Genotype	N (%)	Allele	N (%)
CYP3A5*3 rs776746 (N = 145)	*3/*3	69(47.6)	*3	199(68.6)
	*1/*3	61(42.1)	*1	91(31.4)
	*1/*1	15(10.3)		
CYP3A4*1G rs2242480 (N = 142)	*1/*1	54(38)	*1	176(62)
	*1/*1G	68(47.9)	*1G	108(38)
	*1G/*1G	20(14.1)		
CYP3A4*1B rs2740574 (N = 141)	AA	126(89.4)	A	267(94.7)
	AG	15(10.6)	G	15(5.3)
*ABCB1* C3435T rs1045642 (N = 144)	CC	21(14.6)	C	102(35.4)
	CT	60(41.7)	T	186(64.6)
	TT	63(43.8)		
*ABCB1* G2677T rs2032582 (N = 142)	GG	19(13.4)	G	102(35.9)
	GT	64(45.1)	T	182(64.1)
	TT	59(41.5)		

We observed no statistically significant differences in the tacrolimus C_0_/D ratio between men and women (*p* > 0.5 at all timepoints). Similarly, we observed no significant association between patients’ age and tacrolimus C_0_/D ratio at any timepoint.

### Association of SNP Genotypes With Tacrolimus C_0_/D

We tested the association of the five SNPs with tacrolimus C_0_/D at different time points after transplantation. Of these, CYP3A5*3 and CYP3A4*1G showed strong associations with tacrolimus C_0_/D at all three time points after transplantation (Table 4). Tacrolimus C_0_/D was highest in patients with homozygous *CYP3A5* *3/*3 genotype (non-expressors) compared to CYP3A5*1/*3 and *CYP3A*5 *1/*1 genotypes (expressors) (*p* < 0.001). Among the CYP3A4*1G genotypes, C_0_/D of the patients with *CYP3A4* *1/*1 was highest (*p* < 0.001). CYP3A4*1B AA genotype showed a marginal association with higher C_0_/D on post-operative day 6 (*p* = 0.048). The *ABCB1* variants did not demonstrate a significant association with C_0_/D at any of the post-transplant time points.

**TABLE 4:** Association of SNP genotypes with tacrolimus C_0_/D.

	6th day			6 months			1 year		
**Gene/genotype**	**n**	**C_0_/D**	***p* value**	**n**	**C_0_/D**	***p* value**	**n**	**C_0_/D**	***p* value**
CYP3A5*3									
*3/*3	50	145.45 ± 54.99	<0.001^a^	43	163.06 ± 74.29	<0.001^a^	32	176.1 ± 64.65	<0.001^a^
*1/*3	33	77.1 ± 38.43		38	107.38 ± 50.61		28	114.07 ± 57.21	
*1/*1	9	66.46 ± 36.09		8	87.18 ± 53.18		6	62.38 ± 33.75	
*CYP3A5* Non-expressor	50	145.45 ± 54.99	<0.001^a^	43	163.06 ± 74.29	<0.001^a^	32	176.1 ± 64.65	<0.001^a^
CYP3A5 Expressor	42	74.82 ± 37.77		46	103.87 ± 51.04		28	104.95 ± 57.01	
CYP3A4*1G									
*1/*1	39	146.11 ± 58.02	<0.001^a^	33	168.89 ± 72.7	<0.001^a^	26	180.48 ± 63.39	<0.001^a^
*1/*1G	38	92.65 ± 49.31		41	109.5 ± 58.99		31	121.37 ± 60.75	
*1G/*1G	13	72.02 ± 38.72		13	108.25 ± 55.88		8	66.21 ± 29.39	
CYP3A4*1B									
AA	80	117.36 ± 60.12	0.048^a^	81	132.12 ± 71.19	0.972	63	137.28 ± 70.85	0.472
AG	9	75.87 ± 44.56		5	133.26 ± 55.7		1	189	
***ABCB1* C3435T**									
CC	10	142.57 ± 71.69	0.193	12	125.08 ± 79.03	0.73	11	106.66 ± 56.38	0.216
CT	38	115.5 ± 54.5		33	140.33 ± 72.36		23	151.03 ± 69.46	
TT	43	104.97 ± 60.14		43	129.2 ± 66.65		32	142.39 ± 73.43	
***ABCB1* G2677T**									
GG	8	147.86 ± 71.44	0.219	10	149.08 ± 75.37	0.706	8	133.32 ± 60.61	0.727
GT	42	107.75 ± 55.52		35	127.95 ± 74.06		28	146.16 ± 72.68	
TT	39	111.87 ± 61.19		41	131.69 ± 66.48		28	131.38 ± 72.58	

a Statistically significant.

*CYP3A4* and *CYP3A5* genes are both located in 7q21.1. We found a moderate degree of linkage disequilibrium between CYP3A5*3 (rs776746) and CYP3A4*1G (rs2242480) polymorphisms (D’ = 0.922, *r* ^2^ = 0.64).

### Personalized Initial Dosing Equation Based on Genotype, Age, and Gender

We performed a linear regression analysis to find the association of 6th-day tacrolimus C_0_/D ratio with multiple factors, including CYP3A5*3, CYP3A4*1G, CYP3A4*1B, *ABCB1* C3435T, *ABCB1* G2677T genotypes, age, and gender (Table 5). A dosing equation to calculate the required tacrolimus dose/kg to attain the desired target tacrolimus level during the initial post-transplant period was built using this linear regression (Eq. 1). For uniformity, tacrolimus trough levels of all the patients on day 6 post transplantation were used for tacrolimus initial dose calculation.

**TABLE 5:** Linear regression analysis to find independent predictors of 6th day tacrolimus C_0_/D ratio.

Variable	Coefficient (95% CI)	*p* Value
Constant	158.74(103.01–214.46)	<0.001^a^
Age	−0.36(−1.41–0.69)	0.495
Male gender	3.95(−22.19–30.1)	0.764
*CYP3A5* ^a^3	−40.48[−68.12–(−12.83)]	0.005^a^
*ABCB1* C3435T	−14.48(−40.56–11.59)	0.272
*ABCB1* G2677T	12.66(−14–39.34)	0.348
*CYP3A4* ^a^1G	−11.54(−37.41–14.31)	0.377
*CYP3A4* ^a^1B	−14.66(−51.97–22.64)	0.436

R square = 0.353; *p* value <0.001.

a Statistically significant.

Equation 1 Dosing equation to calculate starting tacrolimus dose/kg to attain optimal initial post-transplant period tacrolimus level

 Required tacrolimus dose/kg=Desired tacrolimus level on Day 6159−(40×CYP3A5 genotype) (1)

*CYP3A5* genotype = 0 for *3/*3, 1 for *1/*3, and 2 for *1/*1.

### Association of *CYP3A5* Expressor Status With Drug-Induced Adverse Effects and Rejection Episodes

The mean nadir serum creatinine was 1.2 ± 0.37 mg/dl. The mean serum creatinine levels at the end of 6 months and 1 year were 1.41 ± 0.58 mg/dl and 1.44 ± 0.74 mg/dl respectively. We did not find any association of any of the SNPs with renal allograft function at the end of 1 year. In our study population, 22 (15.2%) patients developed post-transplant erythrocytosis within a year. Sixty-seven patients (46.2%) had new onset of diabetes mellitus (NODAT) within the 1st year of transplantation. Biopsy proven tacrolimus toxicity was observed in 40 (28%) patients. 24.1% of patients developed graft rejection within a year. A total of 19 (13.1%) patients had biopsy-proven acute graft rejection within first week of transplant. Sixteen patients (11%) had delayed graft function.

The genetic associations of NODAT, renal allograft rejection, and tacrolimus toxicity are summarized in Table 6. *CYP3A5* non-expressors (*3/*3 genotype) had a 2.22-fold higher risk of developing NODAT compared to expressors (*1/*1 + *1/*3 genotypes) (*p* = 0.018, 95% CI = 1.14–4.33). *CYP3A5* expressors had a 2.43 times higher chance of developing rejection within a year of transplantation (*p* = 0.028, 95% CI = 1.08–5.44). *CYP3A5* expressor status showed a trend towards association with biopsy-proven acute graft rejection within first week of transplant (*p* = 0.052, 95% CI = 0.11–1.01). We did not observe a significant association of *CYP3A5* expressor status with the development of tacrolimus toxicity.

**TABLE 6:** Association of CYP3A5 expressor status with post-transplant complications.

Complication	Occurrence	CYP3A5 non-expressor	CYP3A5 expressor	OR (95%CI)	*p* value
NODAT	YES	39	28	2.22 (1.14–4.33)	0.018^a^
	NO	30	48		
Rejection	NO	58	52	2.43 (1.08–5.44)	0.028^a^
	YES	11	24		
Tacrolimus toxicity	YES	22	18	1.51 (0.72–3.15)	0.266
	NO	46	57		

a Statistically significant.

### Global Variation in Allele Frequency Distribution of the Polymorphisms

**FIGURE 1:**
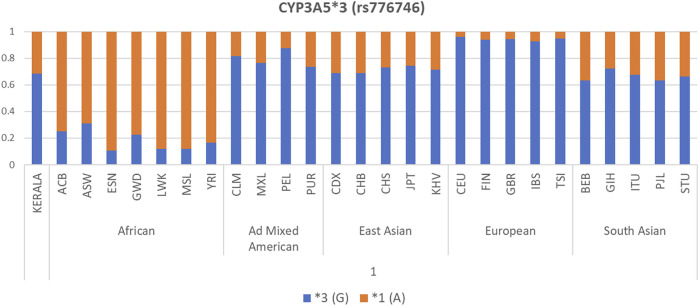
Allele frequency distribution of CYP3A5*3 polymorphism in our Kerala study population compared to 1000 Genomes Project Phase 3 populations.

Allele frequency distribution of the selected *CYP3A5*, *CYP3A4*, and *ABCB1* polymorphisms in our Kerala population and other world populations is presented as bar diagrams in Figure 1 and Supplementary Figure S1. Allele frequencies of drug metabolizer and drug transporter gene polymorphisms in our study population were found to be considerably different compared to other populations worldwide. The CYP3A5*3 (G) allele, which is the major allele in the South Asian (including our study population), Ad Mixed American, East Asian, and European populations was found in very low frequency in African populations.

## Discussion

Therapeutic drug monitoring (TDM) has been an essential and indispensable tool for tacrolimus dosing to control the drug blood concentrations, owing to its narrow therapeutic index and significant inter and intra individual variations in levels. However, since dose modification based on TDM can be done only after the patient is exposed to the drug, the development of a pre-transplant drug level predictive marker is the need of the hour. In our study, we attempted to develop pharmacogenetics-based dose prediction model for initial dosing of tacrolimus in the South Indian population from Kerala.

We investigated the contribution of five polymorphisms in three genes involved in the metabolism and transport of tacrolimus to the dose-adjusted tacrolimus concentration at three different time points. Consistent with the results of earlier studies (Li et al., 2014; Khan et al., 2020; Mendrinou et al., 2020; Hannachi et al., 2021), we found a strong association of CYP3A5*3 polymorphism with tacrolimus C_0_/D at different time points after transplantation (*p* < 0.001). Differential expression of *CYP3A5* is known to influence the tacrolimus bioavailability in individuals. The A > G substitution at nucleotide *6936* in intron 3 of the *CYP3A5* gene, referred to as the *3 allele, results in a splicing defect and formation of a truncated protein that is, not functional, unlike the A nucleotide or *1 allele which is correlated with a high expression of the *CYP3A5* protein. Carriers of one or more copies of the active or wild allele (*1) are *CYP3A5* expressors and have increased tacrolimus clearance. Individuals with homozygous *3/*3 genotype are *CYP3A5* non-expressors (Chen and Prasad, 2018). Apart from *CYP3A5*, the functional SNPs of *CYP3A4* gene may also influence tacrolimus pharmacokinetics. CYP3A4*1G (rs2242480) and CYP3A4*1B polymorphisms are known to have an effect on CYP3A4 enzymatic activity (He et al., 2011; Pączek et al., 2012). Our finding of association of *CYP3A4* polymorphisms with tacrolimus trough levels was also in line with previous reports (Hesselink et al., 2003; Tamashiro et al., 2017). The *CYP3A4* and *CYP3A5* genes are both located in 7q21.1 and the moderate degree of LD between CYP3A4*1G (rs2242480) and the functional variant CYP3A5*3 (rs776746) found in our study might also have an influence on the effect of CYP3A4*1G on tacrolimus C_0_/D. We found no association of *CYP3A4* polymorphisms with tacrolimus C_0_/D in multivariate regression analysis which suggests that the strong association found in univariate analysis could be due to this linkage disequilibrium.

We found no association of polymorphisms in the *ABCB1* gene with tacrolimus level in our population. Prasad et al. (2020), along with an association of *CYP3A5* with tacrolimus level and dose requirement, reported association of *ABCB1* G2677T/A polymorphism with tacrolimus level, dose requirement and P-gp expression in North Indians. They also observed a combined effect of these polymorphisms on tacrolimus dose requirement. Studies on the association of *ABCB1* polymorphisms with tacrolimus pharmacokinetics have yielded inconsistent results across different populations. Positive associations have been reported in Turkish (Ciftci et al., 2013), Caucasian (Kravljaca et al., 2016), Chinese (Wei-lin et al., 2006) and Egyptian (Helal et al., 2017) populations. Haufroid et al. (2006) reported a significant effect of *CYP3A5*, but not *ABCB1* polymorphisms on tacrolimus pharmacokinetic parameters in renal transplant recipients from different ethnic groups. The discrepancies observed in these studies may be due to the ethnic differences in ABCB1 genotype and allele frequencies between populations, which might affect the results of genetic association studies (Seven et al., 2014).

In our study, CYP3A5*3 polymorphism emerged as the single most strongly associated factor determining the dose-adjusted tacrolimus concentration in blood. Using this information, we formulated a simple and easy to compute equation that will help the clinician to calculate, the starting tacrolimus dose per kg body weight to be administered to a patient. The equation was developed using multiple linear regression which also took into account the *CYP3A4* and *ABCB1* polymorphisms, age and gender which may have a minor, but vital role in a patient’s tacrolimus concentration. This genotype-based tacrolimus dose calculation may be beneficial in determining the first tacrolimus dose to be given prior to transplantation. This may help in choosing the individualized dose for each patient thereby prevent rejections due to drug under-dosing and adverse effects due to over-dosing.

Several attempts have been made to optimize tacrolimus dosing based on the transplant recipient’s genotype. Haufroid et al. (2006) observed no association of tacrolimus pharmacokinetic parameters with *ABCB1* polymorphisms, but found a very significant effect of *CYP3A5* polymorphism early after the first administration of tacrolimus in a group of patients belonging to different ethnic groups. They provided a strong argument for a doubling of initial dose in patients carrying at least one CYP3A5*1 allele identified by genotyping patients before transplantation. The French Tactique trial (Thervet et al., 2010) found that the initial tacrolimus dosing based on *CYP3A5* genotype led to significantly more patients reaching the target drug range 3 days after the start of treatment compared to typical, bodyweight-based tacrolimus dosing. The Clinical Pharmacogenetics Implementation Consortium (CPIC) (Birdwell et al., 2015) recommended increasing the starting dose by 1.5–2 times the recommended starting dose in patients *CYP3A5* intermediate (*1/*3 genotype) or extensive metabolizers (homozygous *1/*1 genotype), though total starting dose should not exceed 0.3 mg/kg/day. They recommended that TDM should also be used to guide dose adjustments. A new classification and regression tree model was developed by Wang et al., in 2020 to establish the starting dose of tacrolimus based on the *CYP3A5* genotype and hemoglobin values in Chinese renal transplant recipients. Our results do not contradict these previous attempts to formulate guidelines for genotype-based tacrolimus dosing.

We found that *CYP3A5* expressors (*1/*1 + *1/*3) had an increased chance of rejection than non-expressors (*3/*3). *CYP3A5* expressors, due to their high tacrolimus clearance, have low drug trough concentrations, which may lead to inadequate immunosuppression resulting in rejection. Achieving target blood tacrolimus concentrations during the early post-transplantation period is critical in preventing rejection and improving graft survival. Our findings will help in early identification of patients at a higher risk of developing rejection and possibly prevent rejection by strengthening their immunosuppression.

NODAT, defined as the development of diabetes for the first time after transplantation is a common undesired consequence following solid organ transplantation. It is associated with reduced patient and graft survival and an increased cardiovascular risk (Kasiske et al., 2003; Cosio et al., 2005; Pham et al., 2011). The prevalence of NODAT in solid transplant recipients has been reported to vary from 2 to 53% (Choudhury et al., 2019). We observed a high prevalence of NODAT (46.2%) in our study population, which was not quite unexpected, given Kerala’s high incidence of type 2 diabetes mellitus (21.9%) (Vijayakumar et al., 2019). We found that *CYP3A5* non-expressors had an increased risk of developing NODAT than expressors (*p* = 0.018), owing to their higher tacrolimus bioavailability. The calcineurin inhibitors, tacrolimus and cyclosporine are known to have diabetogenic effects (Heisel et al., 2004). Tacrolimus have been reported to be associated with a higher risk for impaired glucose tolerance (IGT) and NODAT compared to cyclosporine (Reisæter and Hartmann, 2001; Gourishankar et al., 2004). Early identification of patients at a higher risk of developing NODAT may help mitigate NODAT by lifestyle and pharmacological interventions.

Our observation of strong inter-population variations on comparing allele frequencies of *CYP3A5, CYP3A4*, and *ABCB1* gene polymorphisms in our South Indian study population with 1000 genomes Phase 3 populations belonging to different ethnic groups shows that our population is unique with respect to the allele frequency distribution. These inter-ethnic differences in allele frequencies of drug metabolizer and transporter genes emphasize the importance of formulating population-specific dose prediction algorithms based on these gene polymorphisms to draw results of clinical relevance. Population-specific genetic backgrounds should also be taken into account while carrying out pharmacogenetic analyses and clinical trials.

To summarize, we developed a multiple linear regression model-based equation specific to the South Indian population from Kerala to calculate the initial tacrolimus dose/kg to attain optimal initial post-transplant period tacrolimus level. Genotype-guided initial tacrolimus dosing would help transplant recipients achieve optimal tacrolimus levels and thus prevent the adverse effects due to overdose and rejection due to inadequate dose. We envision to carry out further randomized control trial based on this genotype-dependent dosing for tacrolimus efficacy and toxicity minimization. We also found that *CYP3A5* expressors had an increased chance of rejection than non-expressors and non-expressors had an increased risk of developing NODAT than expressors. Our findings will help the clinicians to identify patients at a higher risk of developing rejection and NODAT at an early stage and possibly prevent these by pharmacological interventions and lifestyle modifications.

## Data Availability

The raw data supporting the conclusion of this article will be made available by the authors, without undue reservation.
