# Supplementary figures and images for: Pharmacogenetics Based Dose Prediction Model for Initial Tacrolimus Dosing in Renal Transplant Recipients
Source: Front Pharmacol. 2021 Nov 30;12:726784. doi: 10.3389/fphar.2021.726784 (PMC8669916; doi:10.3389/fphar.2021.726784)

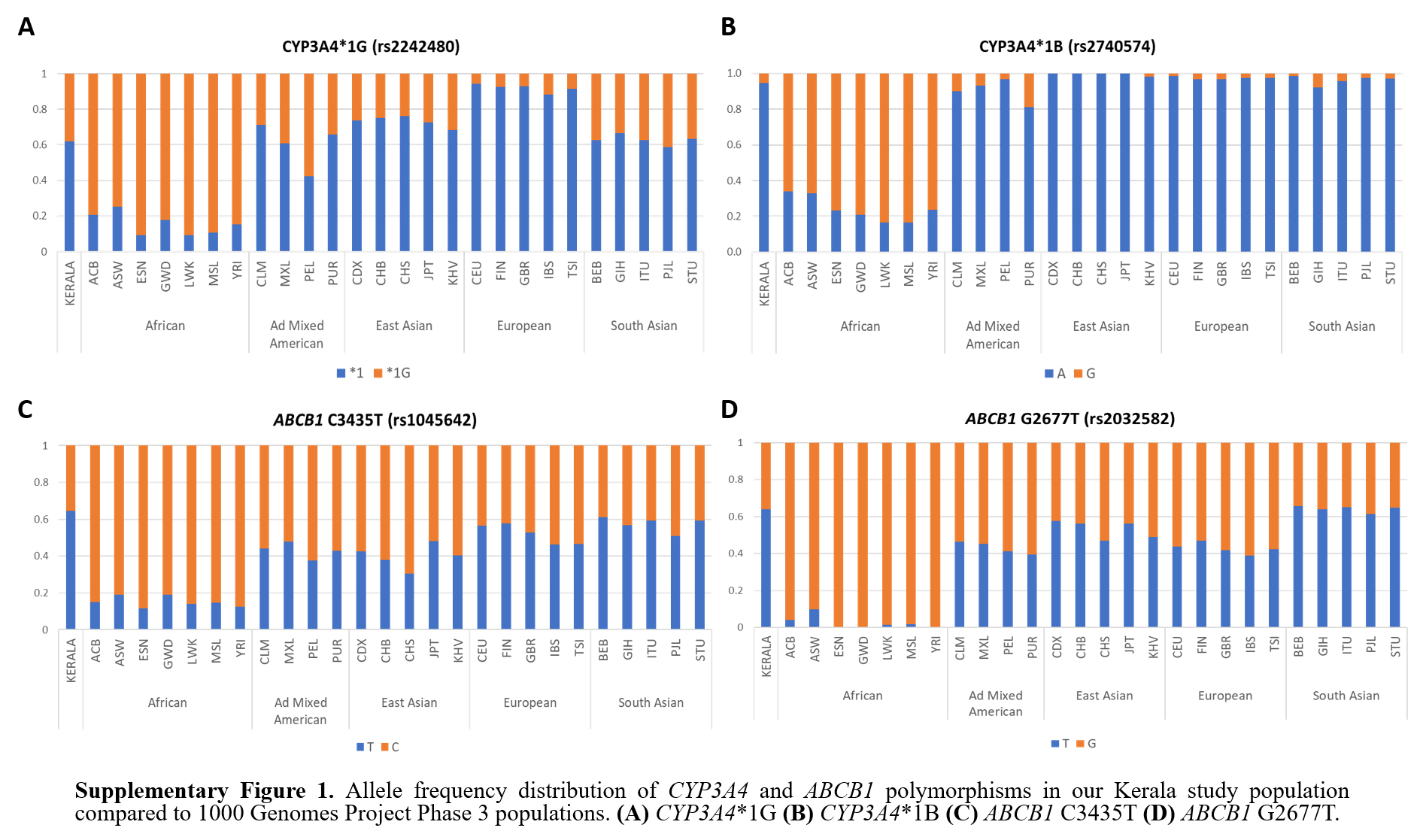

Supplement: Supplementary file 1 [file Image1.TIF]
