# Supplementary material for: Pharmacogenetics Based Dose Prediction Model for Initial Tacrolimus Dosing in Renal Transplant Recipients
Source: Front Pharmacol. 2021 Nov 30;12:726784. doi: 10.3389/fphar.2021.726784 (PMC8669916; doi:10.3389/fphar.2021.726784)
Supplement: Supplementary file 2 [file DataSheet1.pdf]

Srinivas L, Gracious N and Nair RR (2021). Pharmacogenetics based dose prediction model for initial tacrolimus dosing in renal transplant recipients.

Supplementary Tables

Supplementary Table 1. Primer sequences, annealing temperature and restriction enzymes used for SNP genotyping by PCR-RFLP

SNP	Primer Sequence	Annealing temperature	Amplicon size (bp)	RE	Genotypes: fragments (bp)
***CYP3A5**3** **(rs776746)**	F: 5'-CATGACTTAGTAGACAGATGAC-3'<br>R: 5'-GGTCCAAACAGGGAAGAAATA-3'	55°C	293	SspI	*1/*1 (AA): 148+125+20<br>*3/*3 (GG): 168, 125<br>*1/*3 (AG): 168+148+125+20
***CYP3A4**1G** **(rs2242480)**	F: 5'-CACCCTGATGTCCAGCAGAAACT-3'<br>R: 5'-AATAGAAAGCAGATGAACCAGAGCC-3'	62°C	287	RsaI	*1G/*1G (AA): 287<br>*1/*1 (GG): 217+70<br>*1/*1G (AG): 287+217+70
***CYP3A4**1B** **(rs2740574)**	F: 5'- GGAATGAGGACAGCCATAGAGACAAGGGGA -3'<br>R: 5'-CCTTTCAGCTCTGTGTTGCTCTTTG-3'	60°C	380	MboII	AA: 175+169+41<br>GG: 205+175<br>AG: 205+175+169+41
ABCB1 **C3435T (rs1045642)**	F: 5'-TGTTTTCAGCTGCTTGATGG-3'<br>R: 5'-AAGGCATGTATGTTGGCCTC-3'	61.3^{o}C	197	DpnII	TT: 197<br>CC:158+39<br>CT: 197+158+39
ABCB1 **G2677T (rs2032582)**	F: 5'-TGCAGGCTATAGGTTCCAGG-3'<br>R: 5'-TTTAGTTTGACTCACCTTCCCG-3'	53.2^{o}C	224	BanI	TT: 224<br>GG: 198+26<br>GT: 224+198+26

F, Forward primer; R, Reverse primer; RE, Restriction enzyme; bp, Base pairs; Mismatched nucleotide is underlined

Srinivas L, Gracious N and Nair RR (2021). Pharmacogenetics based dose prediction model for initial tacrolimus dosing in renal transplant recipients.

Supplementary Table 2. Description of 1000 Genomes Project Phase 3 populations used for comparison in the present study

Super Population Code	Population Code	Population Description
**African**	ACB	African Caribbeans in Barbados
	ASW	Americans of African Ancestry in SW USA
	ESN	Esan in Nigeria
	GWD	Gambian in Western Divisions in the Gambia
	LWK	Luhya in Webuye, Kenya
	MSL	Mende in Sierra Leone
	YRI	Yoruba in Ibadan, Nigeria
**Ad Mixed American**	CLM	Colombians from Medellin, Colombia
	MXL	Mexican Ancestry from Los Angeles USA
	PEL	Peruvians from Lima, Peru
	PUR	Puerto Ricans from Puerto Rico
**East Asian**	CDX	Chinese Dai in Xishuangbanna, China
	CHB	Han Chinese in Beijing, China
	CHS	Southern Han Chinese
	JPT	Japanese in Tokyo, Japan
	KHV	Kinh in Ho Chi Minh City, Vietnam
**European**	CEU	Utah Residents (CEPH) with Northern and Western European Ancestry
	FIN	Finnish in Finland
	GBR	British in England and Scotland
	IBS	Iberian Population in Spain
	TSI	Toscani in Italia
**South Asian**	BEB	Bengali from Bangladesh
	GIH	Gujarati Indian from Houston, Texas
	ITU	Indian Telugu from the UK
	PJL	Punjabi from Lahore, Pakistan
	STU	Sri Lankan Tamil from the UK
